# Temporal dynamics of angiogenesis: the emerging role of mechanoregulated pathways

**DOI:** 10.1042/BST20253048

**Published:** 2025-08-04

**Authors:** Shayan Zarin-Bal, Margot Passier, Katie Bentley, Tommaso Ristori

**Affiliations:** 1 Department of Biomedical Engineering, Eindhoven University of Technology, Eindhoven, 5612 AZ, The Netherlands; 2 Institute for Complex Molecular Systems (ICMS), Eindhoven University of Technology, Eindhoven, 5612 AZ, The Netherlands; 3 The Francis Crick Institute, London NW1 1AT, U.K; 4 Department of Informatics, King’s College London, London, WC2B 4BG, U.K

**Keywords:** angiogenesis, cell–cell signaling, computational simulations, mechanical cues, mechanosensitivity, tip–stalk selection

## Abstract

Controlling the formation of new blood vessels, i.e. angiogenesis, is a critical challenge for the success of regenerative medicine. The development of effective strategies is hindered by our incomplete understanding of the dynamic mechanisms involved. During physiological angiogenesis, endothelial cells ensure the formation of a functional vascular network by organizing into phenotypic patterns of tip and stalk cells, as mediated by cell–cell signaling communication. While fundamental research identified the major signaling pathways involved in the tip–stalk selection process, recent studies have highlighted the importance of the temporal dynamics of these signaling pathways in determining the final vascular network topology. In this review, we discuss research studies where synergistic approaches between experimental and computational methods led to a renovated understanding of angiogenesis by revealing new temporal regulators of tip–stalk selection. Next, we present increasing evidence suggesting that mechanical cues, such as extracellular matrix stiffness, cyclic strain, and shear stress, are potential temporal regulators of the dynamics of tip–stalk selection and angiogenesis. Future research focused on this promising direction could enable the development of novel approaches that leverage temporal variations of mechanical cues to steer blood vessel growth.

## Introduction

To ensure survival and functionality, most cells in our bodies need to be located within 100–200 µm from blood vessels that facilitate the essential transport of oxygen and nutrients [1]. Lack of oxygen triggers angiogenesis, the biological process leading to the formation of new blood vessels from pre-existing vessels [2]. Controlling and understanding this angiogenic process is crucial not only to treat aberrant vasculature arising across many diseases [3], such as cancer [4,5], retinopathy [6], endometriosis [7], and myocardial infarction [8], but also to develop relatively large tissue-engineered constructs [9,10] and organoid models for disease and drug testing [11,12]

**Figure 1:**
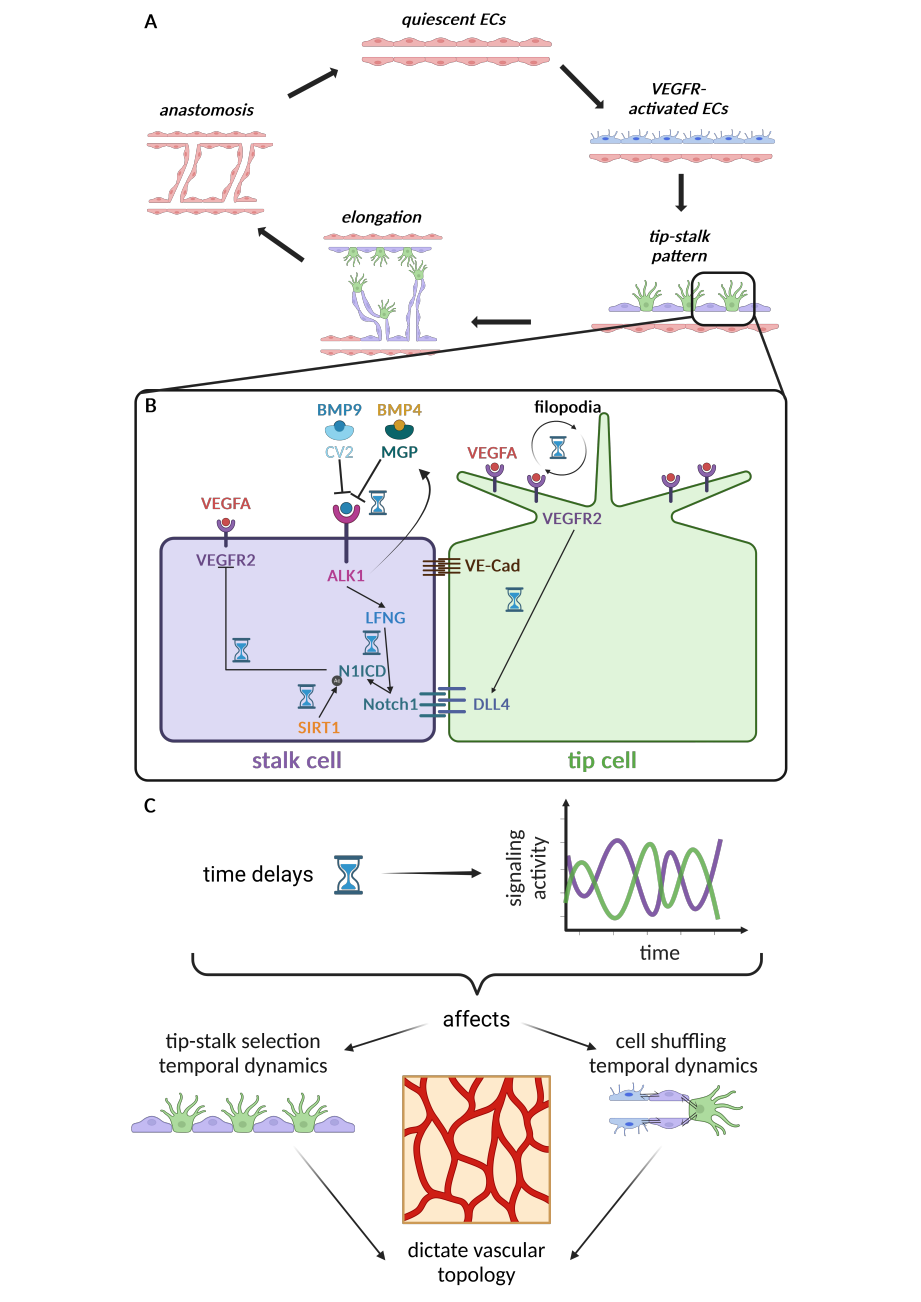
Temporal dynamics of angiogenesis driven by cell-cell signaling. Angiogenesis is characterized by iteratively repeating phases that are tightly organized over space and time (**A**). During these phases, ECs co-ordinate and compete via cell–cell signaling pathways that determine the dynamic patterning of EC selected to acquire the tip or stalk cell behavior (**B**). The cross-talk between these pathways and the delays in between the different signaling phases create short- (over hours) and long-term (over days) fluctuations in the respective signaling activities that in turn affect the temporal dynamics of tip–stalk selection and shuffling, which in turn strongly influence the final vascular topology (**C**). ALK1, activin receptor-like kinase 1; BMP, bone morphogenic protein; CV2, Crossveinless-2; DLL4, delta-like ligand 4; EC, endothelial cell; MGP, matrix gamma-carboxyglutamic acid protein;N1ICD, NOTCH1–intracellular domain; NOTCH1, ; SIRT1, Sirtuin-1; VE-cad, vascular endothelial cadherin; VEGFA, vascular endothelial growth factor A; VEGFR, vascular endothelial growth factor receptor.

Angiogenesis is characterized by iteratively repeating phases, going from angiogenic sprout branch initialization from a perfused vessel to elongation, lumenization, and anastomosis (Figure 1A) [3]. These processes repeat until vessels stabilize with blood flow, pericyte recruitment, and the subsequent reduction in tissue hypoxia signals due to oxygenation [13–15]. During each of these phases, endothelial cells (ECs) composing the new vessels tightly co-ordinate in a dynamic fashion, forming spatiotemporally ordered phenotypic patterns that determine the final vascular network topology (Figure 1) [16–18]. In particular, sprout initialization is characterized by the phenotypic selection of spatially segregated migratory tip cells, which lead the nascent sprout branches, alternated by stalk cells that trail the tip cells and form the sprout stalk [16,19] (Figure 1A and B). This spatial organization of EC phenotypes is usually referred to as tip–stalk selection [20], a phenomenon that occurs in the order of a few hours (less than 12 hours in zebrafish) [21,22]. This tip–stalk pattern changes over time in a dynamic fashion: ECs within sprouts and the forming network continually rearrange positions and switch their tip–stalk phenotypes [23], a phenomenon known as cell shuffling [2,16,20,23–25], which has been observed to occur approximately every 4–6 hours *in vitro*[25]. During anastomosis, the tip cells fuse connecting the new blood vessels, thereby enabling blood flow through the new vessel loop and consequentially improving local tissue oxygenation. This decreases growth factor release and induces EC quiescence, additionally supported by the recruitment of mural cells that sustain vessel stabilization and maturation [26]. The final vascular network topology is established through the regression of poorly perfused vessels, a process known as pruning [14,27].

Given the continual dynamicity and concurrency of these processes, the regulation of EC phenotypic selection and their switching as influenced by temporally changing cross-talking processes is an exciting, emerging new area of study. Several such temporal regulators have now been identified as key determinants of the resulting vascular network branch spacing, width, cellularity, and overall topology [22,28–31]. Elucidating the mechanisms underlying the temporal dynamics of tip–stalk patterning can, therefore, lead to new targets for the control of angiogenesis and treatment of related medical conditions. In this review, we highlight the first discoveries and recent trends identifying temporal regulators of EC phenotypic switches, which were possible due to the combination of experimental methods with predictive simulations (discussed in the second and third sections, respectively). We then highlight the new emerging role of mechanical cues as novel temporal regulators of tip–stalk patterning, before concluding with a discussion on promising future directions.

### Temporal regulators of distinct cell signaling processes determine short- and long-term EC pattern fluctuations

It is well established that EC tip–stalk patterns are regulated by many cross-talking pro- and anti-angiogenic signaling pathways. However, how the timing and dynamics of these pathways affect angiogenesis was only recently investigated. Each of these pathways has corresponding timings and delays, influenced by multiple layers of regulators that interact among each other (Figure 1B). These interactions give rise to positive and negative feedback loops that (Figure 1B), in turn, cause temporal fluctuations of signal activity and angiogenic EC phenotypes [21,32–34] (Figure 1C). While seminal work mainly focused on identifying the key pathways governing these EC phenotypes [19,20,35–37], more recent studies have analyzed the proteins and processes that regulate the different feedback loops, uncovering mechanisms that determine temporal fluctuations in the signaling dynamics that span hours [31,38,39] or days [40], which we, respectively, define as short- and long-term fluctuations.

Fundamental work identified the cross-talk between vascular endothelial growth factor (VEGF) and Notch signaling as critical for tip–stalk selection and shuffling [19,24,25,28,29,36,41–43] (Figure 1B). In general, while VEGF activity promotes the tip cell phenotype inducing migratory behavior through filopodia formation enhancement [20,24,44,45] and VE-cadherin junction weakening [43,46,47], this process is counteracted by stalk-inducing Notch activation. This balance is mediated by the VEGF–Notch cross-talk, which induces a lateral inhibition process and an alternating tip–stalk pattern. In particular, vascular endothelial growth factor receptor 2 (VEGFR2) activation also up-regulates the expression of the Notch ligand, delta-like ligand 4 (DLL4) [20]; the consequential NOTCH1 activation in adjacent cells leads to their down-regulation of VEGFR2–3 expressions and up-regulation of the decoy receptor VEGFR1, making Notch-activated cells less VEGF-sensitive and more inclined to a proliferative stalk phenotype [19,36]. The lateral inhibition process, therefore, establishes the alternating tip–stalk pattern that is present at the onset of angiogenic sprouting and that accompanies EC shuffling at the sprout tip [45]. This latter phenomenon is potentiated by the VEGF–Notch temporal fluctuations arising from their delays and cross-talk (Figure 1B and C), which strongly influence the vascular topology. Experimentally confirmed simulations showed that fluctuations that are non-synchronous among cells promote shuffling and physiological sprouting [31,41]. In contrast, high DLL4 expression or high VEGF conditions, present, for example, in cancer and retinopathy, lead to synchronization of the fluctuations among neighboring ECs and to consequential excessive vessel expansion [31,39,41]. These findings, therefore, highlight the importance of EC pattern temporal dynamics for angiogenesis in health and disease.

As a consequence of these discoveries, more recent work researched for specific regulators of these temporal dynamics, analyzing specific signaling processes affecting Notch signaling, such as protein degradation and ligand–receptor binding [21,30,38,40,48,49]. For example, previous studies have shown that the binding of tissue-derived Semaphorin 3E to Plexin-D1 expressed on ECs down-regulates the expression of DLL4 [50], thereby accelerating tip–stalk selection and the frequency of shuffling events [28]. Similarly, deacetylation of cleaved NOTCH1–intracellular domain (N1ICD) via Sirtuin-1 (SIRT1) was shown to decrease the degradation rate of N1ICD in ECs [38], leading to a prolonged amplitude of NOTCH1 activity, a consequentially longer retention of a stalk-cell phenotype, and therefore impaired vascular expansion and branching [38,39]. Similar roles may be played by other proteins regulating N1ICD degradation rate, such as the ubiquitin-specific peptidase 10 [48] and cyclin-dependent kinase 1 [51,52]. These research directions can also lead to reinterpretation of established research from a different angle. For example, it is well established that angiogenesis is affected by Lunatic Fringe (LFNG) [35], an enzyme that glycosylates NOTCH1 and thereby increases its binding rate to DLL4 [26,35,53]. We have recently shown, using predictive modeling and experiments, that altering LFNG expression changes the timing of EC phenotype selection and tip–stalk shuffling rates [30,39]. These effects of LFNG can contribute to explaining not only its previously observed role in angiogenesis [35] but also the inhibitory effects of bone morphogenic protein–activin receptor-like kinase 1 (BMP9–ALK1) signaling [54]. Indeed, via experimentally validated simulations, we showed that LFNG up-regulation consequential to BMP9–ALK1 activation [30,49] mediates the inhibition of BMP9 on angiogenesis and the EC cross-talk with pericytes [30]. Considering the role of BMP9 signaling in diseases such as hereditary hemorrhagic telangiectasia, this adds to the relevance of angiogenesis temporal regulators in health and disease. To conclude, these studies overall suggest that temporal regulators of angiogenesis can be searched among the mechanisms influencing the single processes of the VEGF–Notch signaling cross-talk, which determine short-term signaling fluctuations and the consequential tip–stalk selection dynamics.

Other recent studies have revealed that, alongside these signaling fluctuations in the Notch–VEGF activity occurring over few hours [31], vascular network formation can also be influenced by long-term fluctuations occurring over 24 hours, arising from other signaling feedback loops. The growth factors BMP4 and BMP9 induce the expression of inhibitors of their activity, matrix gamma-carboxyglutamic acid protein and Crossveinless-2, respectively [40]. This mechanism creates negative feedback loops in both pathways and leads to day-long fluctuations in their downstream gene expressions [40]. These fluctuations of BMP4 and BMP9 activity, in turn, determine alternating temporal phases of EC behavior, which favor either phases of tip selection or stalk selection [55]. The importance of these BMP-inhibitor fluctuations is highlighted by the fact that their disruption results in arteriovenous malformations [40]. Sprouting angiogenesis has been shown to contribute to these pathological malformations [56] in concert with other disrupted cell processes [57–59]. These observations [40,56], together with the emerging role of BMP signaling in regulating tip–stalk selection [30], therefore suggest that disrupted EC patterning dynamics may contribute to arteriovenous malformations. The presence of long-term fluctuations in EC signaling that govern tip–stalk dynamics is further supported by the existence of similar days-long fluctuations crucial for vascular homeostasis, such as those observed regarding the sensitivity of ECs to the von Willebrand factor [60].

Concluding, short- and long-term fluctuations in signaling pathways affecting Notch have been shown to be crucial regulators of the early phases of angiogenesis, such as sprout initiation, branching, and elongation, which all influence the final vascular topology. Temporal regulators of these fluctuations have been found by looking at specific processes within the signaling pathways, such as protein binding and degradation, and by analyzing interchained signaling feedback loops. Future studies might adopt similar approaches to investigate the mechanisms and role of signaling fluctuations at the later stages of angiogenesis, i.e. anastomosis and stabilization [14,61]. Zebrafish [62] and *in vitro* models [61] have already been successful in uncovering molecular mechanisms underlying anastomosis and may be adapted to analyze the effects of temporal perturbations of this process, as done previously for sprout initiation [21,22,30]. More importantly, to potentiate the analysis potential, specific promising experiments and research directions may be indicated by simulations performed in advance. For example, recent simulations suggest that the speed of tip cells may influence not only the vessel diameters and number of branches but also the anastomosis density [14]. This ‘model first and ask questions later’ [63] strategy has already proven central to initiate and accelerate this field, as outlined in the following section.

### The crucial role of *in silico* modeling to unravel tip–stalk temporal dynamics

Computer simulations allow us to interrogate nonlinear feedback dynamics and emergent cellular behavioral effects that systematic perturbations to these dynamics bring, with a breadth and depth currently infeasible with experiments alone [64]. Moreover, *in silico* approaches are particularly well suited for studying dynamic processes such as angiogenesis, as they enable integration of multiscale processes, isolation of specific cues, simulation of behaviors across a broad range of time (and length) scales, and generally allow for hypothesis-driven exploration in a highly efficient and controlled manner [65,66]. The studies mentioned above, which identified the first angiogenic temporal regulators, were based on simulation predictions prior to experimental validation [14,21,22,24,28–31,39,41,43,67]. Due to the fundamental roles of collective cell behavior via cell–cell signaling and spatial features of cells in the temporal dynamics of angiogenic sprouting, the main computational techniques that have been adopted are agent-based models (ABMs) and ordinary differential equation (ODE) models [68–72].

ABMs are a bottom-up approach, where specific attributes are assumed for individual agents to describe their behavior and interaction with other agents and their environment. This approach allows the analysis of phenomena at the collective level that result from interactions at smaller scales [73,74]. In the field of angiogenesis temporal dynamics, ABMs are well suited to capture the emergence of unexpected global spatiotemporal dynamics while collections of individual cells or cell components (agents) interact locally. These models are typically more computationally expensive than ODE models but are well suited for accurately describing heterogeneous cell populations and capturing the heterogeneity in the simulated outcomes. Many ABMs have been developed to investigate angiogenesis and related processes in general [75–83]. The field of angiogenesis temporal dynamics was first pioneered by the ABM presented by Bentley et al., termed the ‘MSM’ (MemAgent Spring Model). In this model, each EC is composed of several agents, collectively representing the cell body and multiple extending filopodia moving in a lattice-free space. Each agent has associated levels of VEGF–Notch proteins, which allows for tracking signal interactions, delays, and feedback loops during cell movement [24,41]. This model and its extensions found numerous applications affecting the field, uncovering many of the mechanisms outlined in the section above [22,24,28,31,41,43,67,84–86]. Overall, cells are proposed as active sensors, where each movement and change of shape influences environmental (VEGF) perception, thereby regulating the tip–stalk selection dynamics [29]. A key example supporting this theory is provided by the recent study of Zakirov et al. [22], where analysis of MSM simulations combined with *in vivo* zebrafish imaging highlighted filopodia extension as an accelerator and stabilizer of tip–stalk patterning during initial sprouting [22]. In particular, the extension of VEGFR-rich filopodia allows cells to actively sense VEGF and increases their likelihood of becoming tip cells against less active neighbors, thereby speeding up the Notch–VEGF lateral inhibition process, which would be much slower otherwise. The MSM framework has been rigorously validated through experimental studies and has provided many detailed simulations of dynamic, signaling-mediated EC behavior during sprouting. Nevertheless, the high level of detail of this model may also impose some limitations on its wide applicability, as it necessitates substantial computational resources and presents complexities in application that require specialized knowledge. One possibility to address this challenge would be the development of more accessible computational platforms designed with user-friendly graphical interfaces, allowing researchers to easily set up and run simulations. For example, computational frameworks such as Morpheus [87] and CompuCell3D [88] allow for a wider and simpler adoption of the Cellular Potts model (CPM), a widely used method to simulate collective cell behavior, for example, during angiogenesis. Nevertheless, despite the potential of CPMs in simulating stochastic processes in the tip–stalk cell selection [89], they have not been systematically applied yet to uncover new temporal regulators of angiogenesis. Moreover, the lattice-based methodology of CPMs decreases freedom in terms of possible cell shapes when compared with the MSM, which might have implications in terms of the analysis of active perception features.

ODE models consist of an abstracted system of ODEs that allow for an accurate analysis of cell signaling dynamics by carefully capturing signaling processes such as receptor binding, gene regulation, protein synthesis, and degradation. Although ODEs do not capture cell movement in the same detail as ABMs, they provide a robust, analytically tractable way to interrogate signaling dynamics in isolation of spatial processes. ODEs have been extensively applied to investigate the Notch-driven dynamics underlying cell phenotypic selections in several settings, not only limited to tip–stalk selection[90–98]. ODE simulations, for example, indicate that Notch signaling feedback loops can determine patterning only if cell heterogeneities are also present [99], such as those introduced by heterogeneous contact areas [100] or signaling noise [101], predicted to be key for tip–stalk selection as well. The first explicit study of VEGF–Notch temporal dynamics determining angiogenesis using ODEs successfully tested the potential role of SIRT1 and LFNG as temporal regulators of sprouting angiogenesis [39], with LFNG later validated experimentally [30]. Building on this model, we further predicted BMP9 [30] and yes-associated protein/transcriptional coactivator with PDZ-binding motif (YAP/TAZ) [102] as key regulators of the temporal dynamics of sprouting angiogenesis. Another recent study analyzed the impact of VEGF levels on the spatiotemporal dynamics of tip–stalk selection by combining ODE analysis of VEGF–Notch cross-talk with experiments [103]. The results suggest that increases in VEGF levels, within specific VEGF ranges, increase the number of tip cells and induce neighboring tip cells (disrupted pattern) [103]. In contrast, if the VEGF levels are increased even higher, other previously experimentally validated ABM simulations predict homogenous EC phenotypes synchronously fluctuating over time [31]. These differences could arise not only from the deviation in experimentally used VEGF concentrations but also from the absence of signaling delays in the first model. Previous experimentally validated models indicate that incorporating signaling delays into the equations is necessary to accurately capture the signaling dynamics and fluctuations [31]. Future studies might thus translate ODEs into delay differential equations to carefully represent real signaling dynamics [104].

Recent efforts have focused on combining computational approaches, such as ODEs and ABMs, creating new hybrid computational models [18,105–107] to build on the respective modeling strengths, while minimizing their limitations. In these modeling studies of angiogenesis, while ABMs allowed for considering heterogeneous spatial features, the combination with ODEs enabled modeling the signaling dynamics considering communication between cells and signaling cross-talk over time. In addition to this usual application in the context of angiogenesis, computational epidemiology studies have shown that hybrid methods can also reduce computational costs compared with pure ABMs, without losing accuracy [108–110]. Future computational studies on angiogenesis may therefore take inspiration from these methodologies to expand the model features while maintaining computational efficiency. For example, taking inspiration from [111], we anticipate that simulating a larger cell population may become feasible by confining the ABM to specific spatially limited regions; these regions would interface with surrounding domains where cells are represented via differential equations, thereby enabling a dynamic movement of cells between the detailed and coarse-grained regions.

### Mechanical cues as new temporal regulators of tip–stalk selection

**Figure 2:**
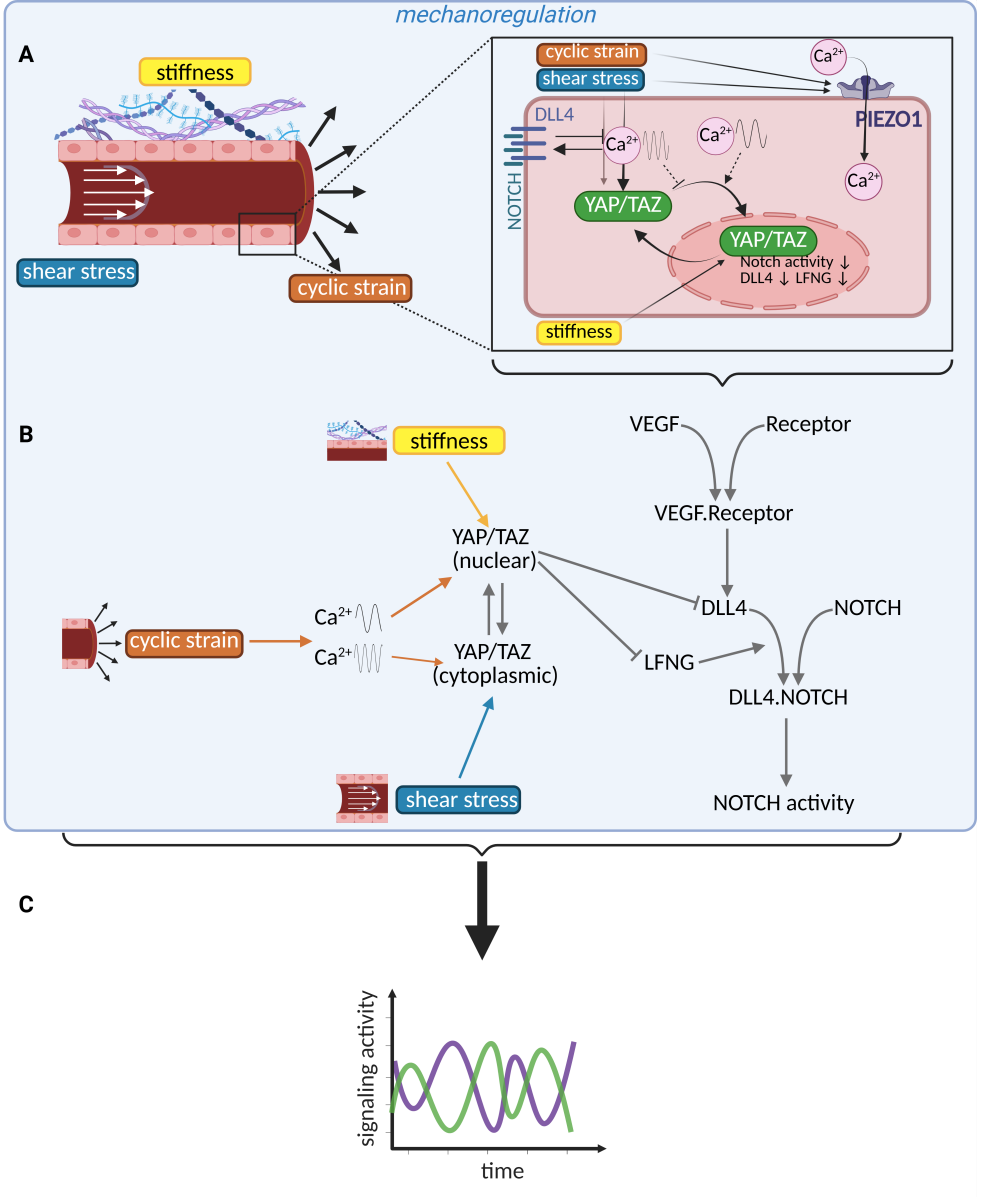
Mechanical cues are potential temporal regulators of tip–stalk selection and angiogenesis. **(A**) Cyclic strain and shear stress trigger PIEZO1 channel activity, causing calcium oscillations and, consequentially, YAP/TAZ nuclearization, which is also influenced by extracellular matrix stiffness. (**B**) Overview of how the mechanical cues affect YAP/TAZ nuclearization and, consequently, the Notch pathway (via DLL4 and LFNG). (**C**) This effect on Notch, in turn, affects the temporal dynamics of signaling activity, dictating the dynamics of phenotypic selection and shuffling, which influence the final vascular network topology. DLL4, delta-like ligand 4; NOTCH, ; PIEZO1, ;TAZ, transcriptional coactivator with PDZ-binding motif; VEGF, vascular endothelial growth factor; YAP, yes-associated protein.

As outlined in the previous section, computational simulations were essential to accelerate the field of (sprouting) angiogenesis and its temporal dynamics. Historically, computational models have also been crucial to analyze the heterogeneous and dynamic mechanical stimuli experienced by cells in complex environments [112–117]. This computational application has the potential to affect the field of angiogenesis temporal dynamics as well. ECs are constantly exposed to a variety of mechanical cues, including stiffness, (cyclic) strain, shear stress, and pressure [118,119]. Mounting evidence shows that these mechanical cues have an impact on angiogenesis [120]. However, the observed effects of shear stress on angiogenesis are contradictory [118,121–124]. Several studies have shown that cyclic and static stretch consistently enhance angiogenic sprouting [125–129], whereas an optimum level seems to exist in terms of extracellular matrix stiffness, with decreased sprouting above or below this threshold stiffness [130–132]. These observations strongly indicate that mechanical cues most likely play a central role in regulating the temporal dynamics of tip–stalk pattern formation. Unraveling the interplay between such (dynamic) mechanical cues and angiogenic signaling pathways could be effectively initiated using *in silico* approaches. Computational simulations would enable isolation of mechanical cues from confounding biochemical noise, as well as allow for systematic exploration of specific combinations of mechanical stimuli. Such computational methods could inform and streamline experimental design, reducing the need for many (redundant) experiments and offering a powerful platform to study intracellular signal integration, such as the mechanoresponsive behavior of angiogenic pathways [63]. Indeed, several angiogenic pathways are known to be mechanoregulated. For example, Notch activity is mechanoregulated in several cells and contexts [133–135]. In ECs, Notch activity decreases with increasing stiffness [136], while it increases with cyclic strain [137]. Interestingly, while Notch activation has been observed in response to relatively high levels of shear stress [138–141], no response in terms of Notch activation was observed for low regimes (Figure 2) [134,138]. Moreover, NOTCH1 is influenced by other mechanosensitive cellular components such as Vimentin [142], YAP/TAZ [143,144], and Cellular Communication Network Factor 1 (CCN1) [133]. Additionally, an interesting avenue to explore could be subcellular forces involved in Notch cleavage and activity, as their regulators could be involved in the regulation of temporal tip–stalk selection dynamics [134,145]. While several computational models [105,146,147] and experiments extensively contributed to elucidate some of the mechanisms involved in angiogenesis mechanoregulation, future studies elucidating the role of mechanical stimuli for the temporal regulation of angiogenesis have the potential to uncover new crucial strategies to control and understand angiogenesis.

In recent computational work, we have taken the first steps in this direction by modeling through ODEs the interaction between Notch and mechanosensitive YAP/TAZ in ECs [102]. Motivated by experiments [144], and by assuming that YAP/TAZ nuclearization [148,149] on stiffer substrates leads to DLL4 and LFNG down-regulation, we could predict that Notch activity decreases for stiffer substrates, for both collective and single cells, consistent with experiments [136]. The simulations also suggested that tip–stalk patterning speed is regulated by the extracellular matrix stiffness through the YAP/TAZ-Notch cross-talk. In particular, we observed a maximal patterning speed at a specific stiffness, with decreased patterning speeds above and below this optimal stiffness, consistent with the angiogenic response observed in previous experiments [29,130–132]. Another experimental study uncovered that ECs dynamically change their environmental stiffness over time [150]. Given the suggested effects of stiffness on EC patterning, this could be an additional mechanism introducing stochasticity in the signaling pathway dynamics [101] and, therefore, potentially influencing the tip–stalk patterning speed. The involvement of YAP/TAZ in the temporal regulation of angiogenesis is further supported by other experimental evidence. Ruehle et al. showed that the time point at which cyclic loading is applied on sprouting ECs influences angiogenesis via YAP-mediated mechanisms, with immediate and delayed loading, respectively, inhibiting and promoting angiogenesis [151]. Given that VEGF enhances YAP/TAZ nuclearization through interactions with the cytoskeleton [152–155] and that such nuclearization is characterized by temporal delays [156], YAP/TAZ temporal dynamics may also mediate the effects that temporally varying dosage of VEGF has on sprouting too [157]. Overall, these studies suggest that mechanosensitive YAP/TAZ may vary over time and regulate the temporal dynamics of EC patterning by affecting Notch.

Besides stiffness, cyclic strain also influences sprouting [158], vessel stabilization [159], and Notch in ECs [137]. This phenomenon may be caused by the effects of cyclic strain on YAP/TAZ, as, for example, observed for MCF10 cells [160]. In addition, the mechanosensitive Ca^2+^-channel PIEZO1 located on the plasma membrane may also play a role. Membrane strain is known to induce activation of PIEZO1, inducing influx of Ca^2+^ ions that in turn influence YAP/TAZ nuclearization as shown in human neural stem cells [161,162]. Computational models integrated with *in vivo* experiments have revealed that the frequency of intracellular Ca^2+^ fluctuations regulates EC tip–stalk selection via a negative feedback loop with the VEGF–Notch pathway [163,164]. This negative feedback loop might be mediated by YAP/TAZ as well, given the influence of YAP/TAZ on Notch proteins [102,144]. It is possible that the observed effects of Ca^2+^-influx on YAP/TAZ nuclearization [160] are dependent on the frequency of Ca^2+^ fluctuations, such that high-frequency Ca^2+^ fluctuations may sequester YAP/TAZ in the cytoplasm, leading to up-regulated DLL4 expression [144] (Figure 2) and to consequentially decreased angiogenesis, consistent with experimental findings [164]. To further investigate this hypothesis, the nuclearization of YAP/TAZ upon cyclic strain in high- and low-frequency Ca^2+^ oscillating conditions should be validated. Unraveling this relationship may provide crucial insights into how Ca^2+^ dynamics regulate YAP/TAZ localization and, consequently, tip–stalk selection in angiogenesis, strengthening the overall hypothesis of seeing mechanical cues and YAP/TAZ as central temporal regulators of tip–stalk selection.

Shear stress is another mechanical stimulus influencing sprouting angiogenesis [120,165,166] and the underlying (mechanosensitive) pathways. This mechanical cue triggers PIEZO1 activation [167] and, in the case of high magnitude, activates Notch signaling in ECs [138,139]. Moreover, high laminar versus oscillatory shear stress, respectively, induces the cytoplasmic and nuclear localization of YAP/TAZ [168], a response likely mediated by the cytoskeleton; while laminar shear stabilizes the cytoskeleton, oscillating flow drives stress fiber polymerization [169,170]. These mechanoregulated mechanisms might strongly influence the tip–stalk dynamics. Existing data indicate that a shear stress threshold at high levels is necessary to induce sprouting in *in vitro* systems [124], suggesting that such a threshold may also be involved in tip–stalk selection. The shear stress levels required to induce angiogenesis correspond closely to those needed to activate Notch signaling and induce YAP/TAZ nuclearization, leading to the hypothesis that a PIEZO1–YAP/TAZ–Notch axis similar to the one hypothesized for the cyclic strain response may also be involved in the angiogenic response to shear stress. Supporting this similarity, analogous to the experiments with time-changing loading [151], it has been shown that the temporal dynamics of shear stress determine YAP/TAZ translocation [168,171], a phenomenon that may be crucial for tip–stalk selection.

Overall, the present literature strongly suggests that mechanical cues are central regulators of the temporal dynamics of tip–stalk selection and angiogenesis, via molecular mechanisms possibly involving the PIEZO1–YAP/TAZ–Notch axis (Figure 2). In this section, we described molecular mechanisms that may explain the mechanoregulation of Notch in ECs and its cross-talk with YAP/TAZ controlling the temporal dynamics of tip–stalk selection. Moreover, we proposed a biological model for the molecular mechanisms guiding the same process in response to cyclic strain and shear stress. In addition to validating this biological model focusing on single mechanical cues at a time, future studies should also investigate the effects of the combination of these cues, since ECs have been shown to respond differently when a combination of mechanical cues is applied [172], providing further insights into the temporal dynamics of EC pattern formation. The validation and extension of the proposed biological models could reveal new strategies to target disease conditions related to angiogenesis and pave the way for new approaches to guide blood vessel growth with mechanics.

### Outlook and conclusion

Gaining a full understanding and control of angiogenesis is essential for the success of regenerative medicine. The process of angiogenesis is highly dynamic, with several pathways and proteins temporally regulating cell behavior and branching events during angiogenesis, affecting the final network topology and functional outcome. We observe a tendency of focusing on the temporal dynamics of initial stages of angiogenesis in recent research, while the temporal dynamics of later phases, such as anastomosis and pruning/remodeling of the formed network, are yet to be studied as these also contribute heavily to the final vascular topology and are affected by mechanical cues [105]. Furthermore, we highlighted that temporal regulators of angiogenesis can be identified by characterizing the dynamic behavior of regulated feedback loops within angiogenic signaling pathways by integrating experimental methods with (hybrid) computational models. We expect that a similar synergy will be crucial to elucidate the role of mechanical cues as key regulators of the spatiotemporal dynamics of angiogenesis.

To potentiate this new research line, *in vitro* systems mimicking the essential characteristics of *in vivo* mechanical cues should be combined with computational simulations. This would enable analysis of complex processes and translation of *in vitro* findings to the *in vivo* conditions. Specifically, hybrid ABM-ODE computational models [18,107] able to replicate the cell–cell signaling among collectively moving cells should be coupled with multiscale finite-element models analyzing the mechanical cues sensed by these ECs [173,174]. Once the simulations suggest targeted experiments [63], already existing platforms could be employed to investigate combined effects of shear stress and cyclic strain [172], cyclic stretch and stiffness [175], or individual bioactive properties such as ECM degradability and stiffness [176,177].

To conclude, we propose that mechanical cues are important (temporal) regulators of patterning angiogenesis, through its effects on the underlying signaling pathways during all phases of angiogenesis, and should therefore be carefully considered in future angiogenic study designs. To accelerate these discoveries, these studies should synergize experiments with simulations, able to analyze the mechanical cues sensed by cells and isolate the complex mechanisms involved. Unraveling such new players may lead to mechanotherapeutic strategies, employing mechanical cues over space and time to gain control over angiogenesis.

**Perspectives**

- The temporal dynamics of endothelial cell (EC) phenotypic selection and angiogenesis heavily influence the final vascular network topology and function. Identifying new temporal regulators of these processes can, thus, lead to the discovery of novel strategies to control angiogenesis for regenerative medicine applications.
- The EC phenotypic selection is a dynamic process mediated by cell signaling pathways. Their signaling cross-talk creates multiple feedback loops central for angiogenesis temporal dynamics. Temporal regulators can thus be identified among the proteins involved in these signaling feedback loops.
- Mounting evidence highlights the several impacts that mechanical cues have on angiogenesis and its underlying signaling pathways. Supported by these recent studies, we propose that mechanical cues are a master regulator of the temporal dynamics of angiogenesis. This promising research direction can lead to the development of new mechanotherapeutic strategies, where mechanical cues are tuned to control blood vessel formation in regenerative medicine.

## Abbreviations

ABM: agent-based model
ALK1: activin receptor-like kinase 1
BMP: bone morphogenic protein
CCN1: Cellular Communication Network Factor 1
CPM: Cellular Potts model
DLL4: delta-like ligand 4
ECs: endothelial cells
LFNG: Lunatic Fringe
MSM: MemAgent Spring Model
N1ICD: Notch1 intracellular domain
ODE: ordinary differential equations
SIRT1: Sirtuin-1
TAZ: transcriptional coactivator with PDZ-binding motif
VEGF: vascular endothelial growth factor
VEGFR: vascular endothelial growth factor receptor
YAP: yes-associated protein 1
